# Survival and treatment in older patients with ewing sarcoma: an analysis of the national cancer database

**DOI:** 10.1186/s40001-023-01599-1

**Published:** 2024-01-05

**Authors:** Marco Braaten, Jacob Braaten, Jasleen Chaddha, Robert Hu, Connor Lanoue, Peter Silberstein, Abubakar Tauseef, Noureen Asghar, Mohsin Mirza

**Affiliations:** 1 Creighton University School of Medicine, Omaha, NE USA; 2 University of Minnesota Medical School, Minneapolis, MN USA; 3 Department of Hematology and Oncology, Creighton University Medical Center, Omaha, NE USA; 4 Department of Internal Medicine, Creighton University Medical Center, Minneapolis, MN USA

## Abstract

**Background:**

Ewing sarcoma (EWS) is a malignancy which primarily arises in adolescence and has been studied extensively in this population. Much less is known about the rare patient cohort over the age of 40 at diagnosis. In this study, we describe the survival outcomes and clinical characteristics of this population.

**Methods:**

This retrospective cohort study utilized the National Cancer Database (NCDB) to identify 4600 patients diagnosed between 2004 through 2019. Of these patients, 4058 were under the age of 40 and 542 were over 40. Propensity score 1:1 matching was performed according to sex and race. Univariate and multivariate logistic regression was performed to generate odds ratios (OR) and a Multivariate Cox regression model was used to generate a hazard ratio (HR) for patients over 40. Kaplan–Meier curves were used to estimate survival from diagnosis to death between age groups. Chi-square tests were used to compare demographic and socioeconomic patient characteristics. IBM statistics version 27.0 was used. *p* < 0.05 was used to indicate statistical significance.

**Results:**

EWS patients older than 40 experienced worse survival outcomes compared to patients under the age of 40. 5-year survival was 44.6% for older patients vs. 61.8% for younger patients (*p* < 0.05). A multivariate Cox proportional hazards model showed that age was independently associated with inferior survival. (HR 1.96; *p* < 0.05). EWS patients over the age of 40 were more likely to have tumors originating from the vertebral column (16.1% vs 8.9%; *p* < 0.05) and cranium (5.3% vs. 2.9%; *p* < 0.05) and had a higher rate of axial tumors (31.6% vs. 18.5%; *p* < 0.05) compared to patients under 40. Additionally, patients older than 40 experienced a significantly longer delay between the date of diagnosis and initiation of systemic treatment (36.7 days vs. 24.8 days; *p* < 0.05) and were less likely to receive adjuvant chemotherapy (93.4% vs. 97.9%; *p* < 0.05).

**Conclusion:**

An age over 40 is associated with decreased survival for patients with EWS. Due to the rarity of EWS in this cohort, the optimal role of systemic treatment remains unknown and has yet to be clearly elucidated. Consequently, our findings suggest that older patients receive disparities in treatment which may be contributing to decreased survival rates.

## Introduction

Ewing sarcoma (EWS) is a family of highly malignant, small cell sarcomas. Following osteosarcoma, it represents the second most common primary malignancy of the bone. EWS classically arises in the diaphysis of lower limb and pelvic bones, but may also occur in extraskeletal sites, most commonly in paravertebral and thoracic soft tissues. EWS disproportionally affects younger patients, with prior literature reporting a median patient age of 15 years at the time of diagnosis [1–3]. With less than ten percent of patients diagnosed with EWS over the age of 40, there exists a paucity of data regarding patients in this cohort [4–9].

Notably, prior literature has reported lower rates of survival in older EWS patients [5, 8–14]. Recently, Liu et al. estimated the 5-year overall survival (OS) at 47.5% for patients over 40 [11]. A 2013 retrospective study by Karski et al. found that patients over 40 experienced significantly decreased survival rates compared to patients less than 40 and were more likely to present with metastatic tumors, as well as tumors of extraskeletal and axial origin [9]. Similarly, other prospective studies have found age to be a significant, independent predictor of mortality and have identified a higher incidence of extraskeletal tumors in older patients [15–17]. However, these prospective studies were largely carried out at single institutions and limited by a small sample size.

There remains a debate in the literature regarding the impact of patient age on survival outcomes, as other studies have found that age is not independently associated with inferior survival [7, 18–20]. For example, a 2008 retrospective study by Pieper et al. analyzed 47 patients over the age of 40 and concluded that with adequate treatment, survival was comparable to that observed in adolescent populations [7]. In addition, a 2000 retrospective study by Bacci et al. found no difference in EWS survival outcomes between the younger cohort (less than 39 years old) and the older cohort (greater than 39 years old) [20].

Surgery, chemotherapy, and radiation are the standard treatments for Ewing sarcoma; however, protocols are not well established for older patients [21]. Previous studies have suggested older patients with EWS may exhibit a poorer response to chemotherapy and are more susceptible to treatment-associated toxicities [22]. Furthermore, the role of treatment differences between old and young EWS patients has never been studied before in a large, retrospective study. Due to the limited and contradictory available data in this population, we sought to provide the largest study to date in this rare population of EWS patients. Using the National Cancer Database (NCDB), we describe patient and tumor characteristics, treatment, and survival data for EWS patients over 40 and compare them to the more common cohort of patients under the age of 40.

## Patients population/method and methodology

### Data sources

This retrospective cohort study utilized the National Cancer Database (NCDB) to identify patients diagnosed with EWS between 2004 and 2019. The NCDB is recognized as the largest existing clinical data registry and includes information on more than 70% of all annual cancer diagnoses in the United States. Patient demographic and outcome data are obtained from over 1500 accredited Commission on Cancer (CoC) facilities and is de-identified by a team of professional registrars. NCDB data are obtained at no cost by applying for a Participant User File (PUF) through the American College of Surgeons in association with Commission on Cancer-accredited cancer programs.

### Data collection and subject selection

This study identified 4864 patients with EWS in the NCDB database using the International Classification of Diseases for Oncology, Third Edition (ICD-O-3), histology code 9260. Out of the 4864 patients who were initially identified, 264 patients were excluded from the study, due to multiple malignancies or missing data. 4600 patients formed the final cohort for statistical Kaplan–Meier and Cox proportional hazard testing.

Patients included in this study were dichotomized into two groups with 40 as cut-off age. Initial demographic analysis was performed with chi-squared and student’s t-tests on patients meeting the inclusion criteria. Differences in insurance status, stage, grade, tumor size, tumor primary site, Charlson-Deyo comorbidity score, median income quartile, and zip code of residence were also reported [23, 24]. Ethnicity was classified into four groups: White, Black, and Asian, and “Other.” Patients of Chinese, Japanese, Filipino, Korean, Thai, Hmong, Vietnamese, Filipino, Korean, Other Asian NOS, Asian NOS, Oriental NOS, Micronesian NOS, and Pacific Islander NOS ethnicity were classified as Asian. American Indian, Aleut or Eskimo, Hawaiian, and Asian Indian or Pakistani NOS was classified as “Other.”

Tumor size was represented as a continuous variable measured in millimeters. Median income was measured using the median household income from 2012–2016 and was classified into four groups as defined by the NCDB participant user file. Charlson-Deyo score was used to assess the patients' comorbidities, and patients were assigned a score of 0, 1, 2, or 3 based on the burden of comorbid disease they carried. Staging at diagnosis was measured using the traditional 0–4 AJCC staging system via the NCDB Analytic Stage Group variable.

### Statistical methods

Propensity score matching was used to create identical 1:1 groups of patients over and under 40 years old matched by sex and race. Chi-Squared and student’s *t*-test were used to perform analysis on demographic differences and clinical outcomes between both groups. Median survival, 5-, and 10-year overall survival and Kaplan–Meier curves were compared by age at diagnosis, and pairwise logrank tests were used to compare the survival distributions. A “crude” multinomial logistic regression model was used to evaluate the association of age over 40 and overall survival. The variables of gender, race, and NCDB analytic stage were then added to the model to generate odds ratios. Finally, a Cox proportional hazard model was constructed to generate a hazard ratio for patients over the age of 40.

All data were analyzed using RStudio using the packages “tidyverse” and “survival.” IBM SPSS Statistics version 25 for Windows, Version 27.0 was also used. *p* < 0.05 was used to indicate statistical significance.

### Ethical considerations

This study utilized participant user files to obtain access to NCDB data through the Participant User Data Files program.

## Results

### Overall characteristics

Of the 4600 patients who met inclusion criteria, 4058 (88.2%) were under 40 and 542 (11.8%) were over 40. Demographic and socioeconomic variables for propensity score-matched and unmatched groups are shown in Table 1. Socioeconomic differences were observed between the older and younger cohorts. Older patients were more likely to be insured with Medicare and come from the highest median income quartile (*p* < 0.05). Clinical characteristics for propensity score-matched and unmatched groups are shown in Table 2. Patients over 40 were also significantly more likely to have higher Charlson-Deyo comorbidity scores at the time of diagnosis (*p* < 0.005).

**Table 1:** Demographic characteristics of EWS patients

Variable	Unmatched		*p*-value	Propensity score (1:1)-matched		*p*-value
	Patients ≤ 40 (*n* = 4058)	Patients > 40 (*n* = 542)		Patients ≤ 40 (*n* = 542)	Patients > 40 (*n* = 542)	
Age (mean (SD))	17.31 (8.09)	54.52 (11.51)	< 0.001	17.65 (8.13)	54.60 (11.49)	< 0.001
Sex, *n* (%)						
Male	2628 (61.4)	351 (60.1)	0.546	351 (50.0)	351 (50.0)	1.000
Female	1652 (38.6)	233 (39.9)		233 (50.0)	233 (50.0)	
Race, *n* (%)						
White	3575 (90.0)	495 (92.4)	0.047	531 (90.9)	531 (90.9)	1.000
Black	127 (3.4)	22 (4.1)		24 (4.1)	24 (4.1)	
Asian	144 (3.6)	11 (2.1)		15 (2.5)	15 (2.5)	
Other	118 (3.0)	8 (1.5)		14 (2.4)	14 (2.4)	
Insurance status, *n* (%)						
Uninsured	177 (4.2)	22 (3.5)	< 0.001	19 (3.3)	22 (3.8)	< 0.001
Private insurance	2847 (66.6)	370 (64.0)		393 (67.3)	370 (63.4)	
Medicaid	989 (22.8)	57 (9.2)		133 (22.8)	57 (9.0)	
Medicare	54 (1.3)	115 (19.6)		8 (1.4)	115 (19.7)	
Other	213 (5.1)	20 (3.7)		31 (5.3)	20 (3.4)	
Median income quartile, *n* (%)						
< $30,000	424 (9.9)	55 (9.4)	0.023	61 (10.4)	55 (9.4)	0.259
$30,000–$34,999	552 (12.9)	58 (9.9)		72 (12.3)	58 (9.9)	
$35,000–$45,999	1938 (45.3)	251 (43.0)		260 (44.5)	251 (43.0)	
≥ $46,000	1366 (31.9)	220 (37.7)		191 (32.7)	220 (37.7)

**Table 2:** Clinical characteristics of EWS patients

Variable	Unmatched		*p*-value	Propensity score (1:1)-matched		*p*-value
	Patients ≤ 40 (*n* = 4058)	Patients > 40 (*n* = 542)		Patients ≤ 40 (*n* = 542)	Patients > 40 (*n* = 542)	
AJCC analytic stage group (%)						
1	422 (9.9)	59 (10.1)	0.128	61 (10.4)	59 (10.1)	0.255
2	1144 (26.7)	172 (29.5)		163 (27.9)	172 (29.5)	
3	94 (2.2)	18 (3.1)		9 (1.5)	18 (3.1)	
4	1055 (24.6)	151 (25.9)		141 (24.1)	151 (25.9)	
Unknown	1565 (36.6)	184 (31.5)		210 (36.0)	184 (31.5)	
Anaplastic (grade)	634 (16.8)	87 (17.4)	0.728	82 (16.0)	87 (17.4)	0.546
Charlson-Deyo score, *n* (%)						
0	3880 (95.6)	458 (84.5)	< 0.001	558 (95.5)	494 (84.6)	< 0.001
1	147 (3.6)	60 (11.1)		23 (3.9)	65 (11.1)	
2	23 (0.6)	18 (3.3)		1 (0.2)	18 (3.1)	
≥ 3	8 (0.2)	6 (1.1)		2 (0.3)	7 (1.2)	
Primary site, *n* (%)						
Long bones of upper limb (C400)	453 (10.6)	52 (8.9)	< 0.001	68 (11.6)	52 (8.9)	< 0.001
Short bones of upper limb (C401)	44 (1.0)	10 (1.7)		10 (1.7)	10 (1.7)	
Long bones of lower limb (C402)	1093 (25.5)	132 (22.6)		149 (25.5)	109 (18.7)	
Short bones of lower limb (C403)	155 (3.6)	14 (2.4)		18 (3.1)	14 (2.4)	
Pelvic bones, sacrum, coccyx (C414)	1032 (25.4)	122 (22.5)		152 (26.0)	132 (22.6)	
Bone of limb, NOS (C409)	43 (1.0)	10 (1.7)		6 (1.0)	10 (1.7)	
Bones of skull and face (C410)	201 (4.7)	31 (5.3)		17 (2.9)	31 (5.3)	
Mandible (C411)	59 (1.4)	9 (1.5)		12 (2.1)	9 (1.5)	
Vertebral column (C412)	422 (9.9)	94 (16.1)		52 (8.9)	94 (16.1)	
Rib, sternum, clavicle (C413)	476 (11.1)	60 (10.3)		67 (11.5)	60 (10.3)	
Overlapping lesions (C408 + C418)	47 (1.1)	13 (2.0)		6 (1.0)	12 (2.0)	
Bone of non-limb, NOS (419)	195 (4.6)	51 (8.7)		27 (4.6)	51 (8.7)	
Appendicular tumor (C400-403, C409, C414)	3075 (71.8) (*app.*)	378 (64.7) (*app.*)	< 0.001	403 (68.9) (*app.*)	327 (56.0) (*app.*)	< 0.001
Axial tumor (C410, C411, C412, C419)	877 (20.5) (*axial*)	185 (31.7) (*axial*)	< 0.001	108 (18.5) (*axial*)	185 (31.6) (*axial*)	< 0.001

### Tumor characteristics

Differences in tumor primary site were observed between the two groups. Tumor and treatment variables for propensity score-matched and unmatched groups are shown in Table 3. Following propensity score matching, EWS patients over the age of 40 were more likely to have tumors of axial primary site (31.6% vs. 18.5%; *p* < 0.05). They also had higher rates of tumors originating from the vertebral column (16.1% vs 8.9%; *p* < 0.05) and cranium (5.3% vs. 2.9%; *p* < 0.05) compared to patients under 40, who were more likely to have appendicular primary site tumors. Older patients had larger tumors compared to younger patients (525 mm vs. 518 mm; *p* = 0.807); however, this difference was not statistically significant. No significant differences in average stage at diagnosis or extent of lymph node metastasis were observed.

**Table 3:** Treatment characteristics of EWS patients

Variable	Unmatched		*p*-value	Propensity score (1:1) matched		*p*-value
	Patients ≤ 40 (*n* = 4058)	Patients > 40 (*n* = 542)		Patients ≤ 40 (*n* = 542)	Patients > 40 (*n* = 542)	
Surgery of primary site, *n* (%)						
Surgery of primary site performed	2102 (51.8)	253 (46.7)	0.025	268 (49.4)	253 (46.7)	0.362
Surgery of primary site not performed	1956 (48.2)	289 (53.3)		274 (50.6)	289 (53.3)	
Surgical margins, *n* (%)						
No residual tumor	1456 (35.9)	146 (26.9)	< 0.001	179 (33.0)	146 (26.9)	0.121
Microscopic residual tumor	96 (2.4)	13 (2.4)		14 (2.6)	13 (2.4)	
Macroscopic residual tumor	47 (1.2)	12 (2.2)		5 (0.9)	12 (2.2)	
Surgical margins unknown	96 (2.4)	16 (3.0)		15 (2.8)	16 (3.0)	
Palliative care *n* (%)						
Received	168 (4.1%)	42 (7.7%)	< 0.001	28 (5.2)	42 (7.7)	0.084
Did not receive	3890 (95.9%)	500 (92.3%)		514 (94.8)	500 (92.3)	
Adjuvant therapy, *n* (%)						
Adjuvant chemotherapy	1826 (96.2%)	198 (93.4%)	0.039	234 (97.9)	198 (93.4)	0.017
Adjuvant radiation	14 (0.7%)	5 (2.4%)		0 (0.0)	5 (2.4)	
Adjuvant chemoradiation	58 (3.1%)	9 (4.2%)		5 (2.1)	9 (4.2)	
Time to surgery (mean days (SD))	99.0 (69.1)	85.6 (89.7)	0.005	102.4 (73.6)	85.6 (89.7)	0.020
Tumor size (mean mm (SD))	510.8 (455.6)	525.7 (459.6)	0.053	518.9 (455.2)	525.7 (459.6)	0.807
Time to initiation of treatment (mean days (SD))	19.2 (29.1)	29.5 (36.7)	< 0.001	18.7 (24.8)	29.5 (36.7)	< 0.001
Time to chemo (mean days (SD))	23.1 (28.4)	46.3 (42.4)	< 0.001	22.8 (26.7)	46.3 (42.4)	< 0.001

### Treatment characteristics

#### Chemotherapy

The two groups experienced differences in treatment type and timing. Propensity score-matched patients older than 40 experienced a significantly longer delay between the date of diagnosis and initiation of systemic treatment (29.5 days vs. 18.7 days; *p* < 0.05). Older patients also received lower rates of adjuvant chemotherapy (93.4% vs. 97.9%; *p* < 0.05) and experienced a marked delay in initiation of chemotherapy (46.3 days vs. 22.8 days; *p* < 0.05) following diagnosis when compared to the cohort under 40.

#### Surgery

Patients over 40 were less likely to undergo a surgical procedure of the primary site in the unmatched group (46.7% vs. 51.8%; *p* < 0.05); however, this finding was not replicated in the propensity score-matched group (46.7% vs. 49.4%; *p* = 0.362). In the propensity score-matched group, older patients experienced a shorter delay between diagnosis and definitive surgical procedure of the primary site compared to patients under 40 (85.6 days vs. 102.4 days; *p* < 0.05). The cohort over 40 were less likely to undergo a clean resection without residual tumor remaining, albeit this difference was not significant between propensity score-matched groups (26.9% vs. 33.0%; *p* = 0.121).

### Overall survival

**Fig. 1:**
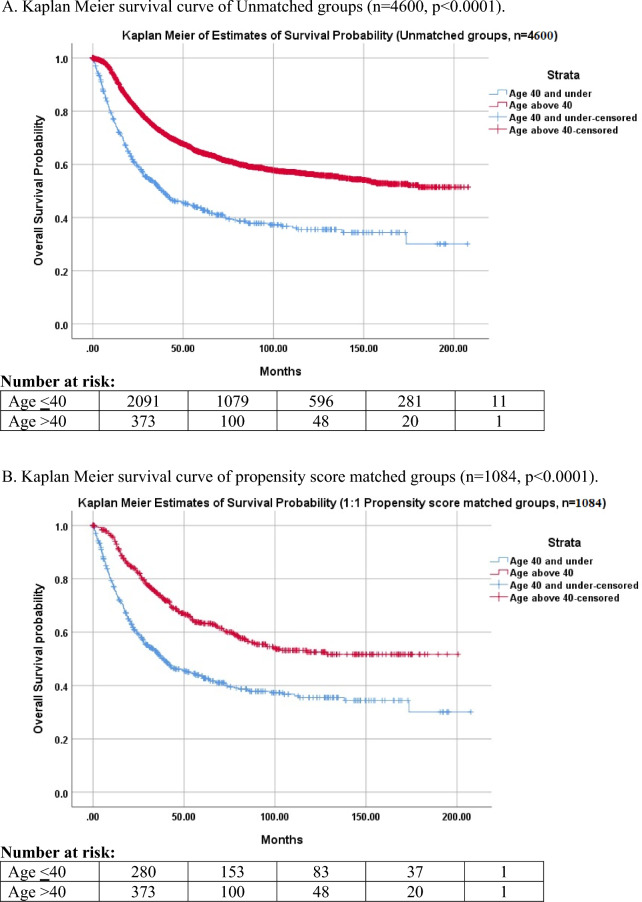
Kaplan Meier estimate of overall survival for EWS patients **A**. unmatched groups, **B** 1:1 propensity score-matched groups

As seen in Fig. 1B of propensity score-matched groups, patients over 40 experienced inferior 5-year (44.6% vs. 61.8%; *p* < 0.05) and median (38.6 months vs. 73.9 months; *p* < 0.05) estimates of overall survival on Kaplan–Meier analysis. Table 4 demonstrates how age over 40 had an odds ratio (OR) of 2.08 [1.61–2.69] (*p* < 0.05) after controlling for gender, race, and NCDB analytic stage. The Cox proportional hazards model results reveal that age over 40 was independently associated with inferior survival and an increased hazard ratio for death of 1.96 [1.64–2.34] (*p* < 0.05) as shown in Table 4.

**Table 4:** Multivariate regression models for association of age > 40 with poor survival

Variables	Unmatched groups *n* = 4600			Propensity score-matched groups (1:1) *n* = 1084		
	Odds ratio (OR)/hazard ratio (HR)	95% confidence interval (CI)	*p*-value	Odds ratio (OR)/hazard ratio (HR)	95% confidence interval (CI)	*p*-value
Crude model (unadjusted)	2.23	1.85–2.66	< 0.001	2.00	1.57–2.56	< 0.001
Model adjusted for gender, race and stage	2.35	1.94–2.84	< 0.001	2.08	1.61–2.69	< 0.001
Cox proportional model for hazard ratio	2.08	1.83–2.35	< 0.001	1.96	1.64–2.34	< 0.001

## Discussion

The main finding of this study is that after adjusting for competing factors, EWS patients over the age of 40 experienced significantly decreased rates of survival compared to patients under 40. Additionally, we found that older patients with EWS exhibited unique clinical and treatment characteristics. Specifically, the cohort over 40 presented with higher rates of axial primary site tumors. They also were less likely to receive adjuvant chemotherapy, and experienced longer delays in receiving treatment following diagnosis compared to patients under 40. Together, these findings imply that increased age is significantly associated with decreased survival rates and that treatment and tumor differences may be contributing to differences in survival.

Our finding of lower survival in patients over the age of 40 is consistent with numerous prior studies which have identified age to be independently associated with inferior survival in patients with EWS [5, 8–14]. Specifically, our results are in accordance with the 2013 analysis of the SEER database by Karski et al*.* as well as a large 2010 study by Lee et al*.* which used the California Cancer Registry, both of which found increased age to be independently associated with inferior survival [9, 17]. However, other, more recent studies have shown that with similar treatment, there is comparative survival between older and younger patients [6, 7, 20, 25]. In the present study, survival differences between age groups were consistently observed in both uni- and multivariate models and after propensity score matching, therefore, we feel age over 40 is likely an independent negative prognostic factor. However, it remains uncertain whether older age may also be serving as a proxy for high-risk features seen disproportionately in older patient populations.

One high-risk feature is tumor volume and later stage at diagnosis. Our study found that older patients over 40 experienced larger tumor size compared to younger patients, but this difference was not statistically significant. Similarly, we found no differences in disease stage at presentation between the two groups. Conversely, Hense et al. analyzed 945 patients and found a significant association between age and tumor volume—a finding shared by Cotteril, who found that patients over the age of 15 had significantly greater tumor size [26, 27]. Therefore, we theorize that there may be other high-risk features of disease in older EWS patients contributing to decreased survival.

Previous studies have identified an axial primary site as a potentially important prognostic factor in patients with EWS [5, 9, 27]. We found that patients over 40 had significantly increased incidence of axial skeleton tumors, specifically of the vertebral column and cranium. They also experienced significantly higher residual macro- and microscopic tumor following resection. Cotteril et al. reported that increased age and axial primary tumor site, specifically of the pelvis, were associated with higher tumor burden, increased propensity for metastasis, and significantly decreased survival rates [27]. Similarly, Karski et al. observed higher rates of axial primary site tumors in the age over 40 cohort and hypothesized that these primary tumor sites may confer a unique tumor pathophysiology and worse prognosis [9]. It is also possible that primary site may guide decisions about surgical treatment, which has been shown to confer the greatest effect on overall survival. This holds true even in the case of challenging primary sites, such as metastatic disease and pelvic tumors [28]. For instance, our study found that older patients experienced lower rates of surgery compared to younger patients and had higher residual tumor burden following resection. However, these differences were no longer statistically significant following propensity score matching; therefore, surgical discrepancies in older patients may be better explained by other clinical features such as race or sex. Increased comorbidity scores were also seen in the older patient group, which may manifest as a tendency for surgeons to direct higher-risk, older patients toward conservative therapy, despite potentially benefiting from surgery.

The optimal role of systemic treatment in older EWS patent populations is currently unknown and remains highly debated. We found that older patients experienced lower rates of adjuvant chemotherapy and significantly delayed initiation of systemic treatment. This finding supports the notion that treatment differences between age groups exacerbating the differences in survival. In a study of 53 patients, Gupta et al. found that adults with EWS were treated with similar doses of chemotherapy—ifosfamide or cyclophosphamide—and local therapy as pediatric patients; however, adults received a longer delay in the initiation of definitive treatment, which was found to negatively impact survival in adult patients [29]. Delayed initiation of therapy for older patients was also seen in the Karski study who hypothesized that older EWS patients may not receive as intensive treatment due to non-standard treatment protocols as compared to younger patients [9]. Because EWS is much less common in patients over 40 years, patient treatment decisions are subject to the discretion of the patient’s multidisciplinary oncology team and do not abide by the standardized 12 week (about 3 months) treatment protocols that exist for pediatric patients.

Contraindications or complications from systemic treatment is another risk factor which may be responsible for the lower rates of chemotherapy observed in the older EWS patient population. Numerous other studies have investigated the relationship between age and chemotherapy toxicity, with conflicting results [10, 20, 30]. Panda et al. analyzed 66 patients over 40 and identified significant rates of toxicity, primarily in the form of peripheral neuropathy from vincristine [10]. Bacci et al. found that older patients (> 39 years old) were more likely to experience significant hematologic malignancies (36% vs. 15%), but found no dose delays between age cohorts and concluded that older patients should be included in multidisciplinary trials with similar treatment regimens [20]. Similarly, Verrill et al. concluded that while IVAD chemotherapy regimens are myelotoxic in adults, they can be given safely and thus should be incorporated into adult regimens [6]. These results suggest that the current ethos of EWS management in older patients may be contributing to the differences in treatment protocol and delivery observed in the present study.

This study was subject to a number of limitations including its retrospective design, absence of cancer specific mortality, and chemotherapy regimen details. The NCDB captures data only from participating institutions, representing ~ 70% of cancer cases in the U.S, which is not necessarily a complete sample [31]. Finally, the NCDB does not contain data on baseline clinical features such as White Blood Cell (WBC) count, albumin, or specific comorbidities for each patient. Prior studies have brought out these parameters as important features of disease; therefore, a limitation of this study was the lack of specific information with respect to these factors [32].

## Data Availability

Data from the National Cancer Database (NCDB) is provided through qualifying institutions. Institutions are required to complete a data use agreement and submit data annually. NCDB data are available through an application process to investigators associated with Commission on Cancer-accredited programs.

## References

[1] Riggi N, Suvà ML, Stamenkovic I (2021). Ewing’s SARCOMA. N Engl J Med.

[2] Burchill S (2003). Ewing’s sarcoma: diagnostic, prognostic, and therapeutic implications of molecular abnormalities. J Clin Pathol.

[3] Davis D, Berg E, Ranjit E, Bhandari P, Sapra A (2020). Clinicians beware, ewing's sarcoma after 60 is elusive and rare. Cureus!.

[4] Siegel RD, Ryan LM, Antman KH (1988). Adults with Ewing's sarcoma. An analysis of 16 patients at the Dana-Farber Cancer Institute. Am J Clin Oncol.

[5] Sinkovics JG, Plager C, Ayala AG, Lindberg RD, Samuels ML (1980). Ewing sarcoma: its course and treatment in 50 adult patients. Oncology.

[6] Verrill MW, Judson IR, Harmer CL, Fisher C, Thomas JM, Wiltshaw E (1997). Ewing's sarcoma and primitive neuroectodermal tumor in adults: are they different from Ewing's sarcoma and primitive neuroectodermal tumor in children?. J Clin Oncol.

[7] Pieper S, Ranft A, Braun-Munzinger G, Jurgens H, Paulussen M, Dirksen U (2008). Ewing's tumors over the age of 40: a retrospective analysis of 47 patients treated according to the International Clinical Trials EICESS 92 and EURO-E.W.I.N.G. 99. Onkologie.

[8] Baldini EH, Demetri GD, Fletcher CD, Foran J, Marcus KC, Singer S (1999). Adults with Ewing's sarcoma/primitive neuroectodermal tumor: adverse effect of older age and primary extraosseous disease on outcome. Ann Surg.

[9] Karski EE, Matthay KK, Neuhaus JM, Goldsby RE, Dubois SG (2013). Characteristics and outcomes of patients with Ewing sarcoma over 40 years of age at diagnosis. Cancer Epidemiol.

[10] Panda G, Chandrasekharan A, Das S (2022). Outcomes of Ewing sarcoma in adults over 40 years of age from a low-middle income country. Ecancermedicalscience.

[11] Liu HF, Wang JX, Zhang DQ, Lan SH, Chen QX (2018). Clinical features and prognostic factors in elderly ewing sarcoma patients. Med Sci Monit.

[12] Wan Z-H, Huang Z-H, Chen L-B (2017). Survival outcome among patients with Ewing’s sarcoma of bones and joints: a population-based cohort study. Sao Paulo Med J.

[13] Wilkins RM, Pritchard DJ, Burgert EO, Unni KK (1986). Ewing's sarcoma of bone. Experience with 140 patients. Cancer.

[14] Craft AW, Cotterill SJ, Bullimore JA, Pearson D (1997). Long-term results from the first UKCCSG Ewing's Tumour Study (ET-1). United Kingdom Children's Cancer Study Group (UKCCSG) and the Medical Research Council Bone Sarcoma Working Party. Eur J Cancer.

[15] Cesari M, Righi A, Cevolani L (2016). Ewing sarcoma in patients over 40 years of age: a prospective analysis of 31 patients treated at a single institution. Tumori.

[16] Rochefort P, Italiano A, Laurence V (2017). A retrospective multicentric study of ewing sarcoma family of tumors in patients older than 50: management and outcome. Sci Rep.

[17] Lee J, Hoang BH, Ziogas A, Zell JA (2010). Analysis of prognostic factors in Ewing sarcoma using a population-based cancer registry. Cancer.

[18] Oberlin O, Habrand JL, Zucker JM (1992). No benefit of ifosfamide in Ewing's sarcoma: a nonrandomized study of the French Society of Pediatric Oncology. J Clin Oncol.

[19] Oberlin O, Deley MC, Bui BN (2001). Prognostic factors in localized Ewing's tumours and peripheral neuroectodermal tumours: the third study of the French Society of Paediatric Oncology (EW88 study). Br J Cancer.

[20] Bacci G, Ferrari S, Comandone A (2000). Neoadjuvant chemotherapy for Ewing's sarcoma of bone in patients older than thirty-nine years. Acta Oncol.

[21] Subbiah V, Anderson P, Lazar AJ, Burdett E, Raymond K, Ludwig JA (2009). Ewing’s sarcoma: standard and experimental treatment options. Curr Treat Options Oncol.

[22] Ferrari S, Bielack SS, Smeland S (2018). EURO-BOSS: a European study on chemotherapy in bone-sarcoma patients aged over 40: Outcome in primary high-grade osteosarcoma. Tumori J.

[23] Ladha KS, Zhao K, Quraishi SA (2015). The Deyo-Charlson and Elixhauser-van Walraven Comorbidity Indices as predictors of mortality in critically ill patients. BMJ Open.

[24] Glasheen WP, Cordier T, Gumpina R, Haugh G, Davis J, Renda A (2019). Charlson Comorbidity Index: *ICD-9* Update and *ICD-10* Translation. Am Health Drug Benefits.

[25] Fizazi K, Dohollou N, Blay JY, Guérin S, Le Cesne A, André F, Pouillart P, Tursz T, Nguyen BB (1998). Ewing's family of tumors in adults: multivariate analysis of survival and long-term results of multimodality therapy in 182 patients. J Clin Oncol.

[26] Hense HW, Ahrens S, Paulussen M, Lehnert M, Jürgens H (1999). Factors associated with tumor volume and primary metastases in Ewing tumors: results from the (EI)CESS studies. Ann Oncol.

[27] Cotterill SJ, Ahrens S, Paulussen M, Jürgens HF, Voûte PA, Gadner H, Craft AW (2000). Prognostic factors in Ewing's tumor of bone: analysis of 975 patients from the European Intergroup Cooperative Ewing's Sarcoma Study Group. J Clin Oncol.

[28] Miller BJ, Gao Y, Duchman KR (2017). Does surgery or radiation provide the best overall survival in Ewing's sarcoma? A review of the National Cancer Data Base. J Surg Oncol.

[29] Gupta AA, Pappo A, Saunders N, Hopyan S, Ferguson P, Wunder J, O'Sullivan B, Catton C, Greenberg M, Blackstein M (2010). Clinical outcome of children and adults with localized Ewing sarcoma. Cancer.

[30] Kaidar-Person O, Haim N, Bar-Sela G (2011). Treatment of adult patients with Ewing’s sarcoma: compliance with chemotherapy protocols & toxicity. Med Oncol.

[31] Bilimoria KY, Bentrem DJ, Stewart AK, Winchester DP, Ko CY (2009). Comparison of commission on cancer-approved and -nonapproved hospitals in the United States: implications for studies that use the National Cancer Data Base. J Clin Oncol.

[32] Sasi A, Ganguly S, Biswas B, Pushpam D, Kumar A, Agarwala S, Khan SA, Kumar VS, Deo S, Sharma DN, Biswas A, Mridha A, Barwad A, Thulkar S, Bakhshi S (2022). Development and validation of a prognostic score at baseline diagnosis for Ewing sarcoma family of tumors: a retrospective single institution analysis of 860 patients. Am J Transl Res.

